# Severe cutaneous adverse reactions in a tertiary care center in Jamaica

**DOI:** 10.1016/j.jdin.2024.02.007

**Published:** 2024-02-19

**Authors:** Alicia J.S. McNish, Jonathan D. Ho, Althea D.C. East-Innis

**Affiliations:** Dermatology Unit, Department of Medicine, University of the West Indies, Kingston, Jamaica

**Keywords:** acute generalized exanthematous pustulosis, drug reaction with eosinophilia and systemic symptoms, severe cutaneous drug reactions, Stevens Johnson syndrome, Stevens Johnson syndrome/toxic epidermal necrolysis overlap, toxic epidermal necrolysis

## Abstract

**Background:**

Severe cutaneous adverse reactions (SCARs) are associated with morbidity and mortality.

**Objective:**

The aim was to determine the different types of SCARs, their morphology, common offending drugs, interventions, and outcomes.

**Methods:**

A retrospective cohort study was conducted of all patients admitted to the dermatology service at the University Hospital of the West Indies with Stevens-Johnson syndrome (SJS), SJS/toxic epidermal necrolysis overlap (TEN), TEN, drug reaction with eosinophilia and systemic symptoms and acute generalized exanthematous pustulosis between January 1, 2012 to June 1, 2022.

**Results:**

Fifty-one cases (51) met the inclusion criteria for SCAR. SJS, SJS/TEN overlap and TEN together accounted for 71.2% of cases. SCARs were most frequent in the fourth, fifth and 6th decades of life and there was a female preponderance. Antibiotics (31%) and anticonvulsants (29%) were the most common causative agents for SCARs. Most patients had at least 1 complication. The liver was the most common extracutaneous organ affected. Mortality was 7.8%. The main cause of death was sepsis.

**Limitations:**

Results were not generalizable. There were missing data and loss to follow-up.

**Conclusion:**

Judicious use of antimicrobials and corticosteroids may be beneficial in treatment of severe cutaneous drug reactions.

**Capsule Summary**

• The article shows that compared to other studies, there are similarities and differences in suspected agents, complications and mortality for some SCARs.
• The article raises awareness that the appropriate use of antimicrobials and corticosteroids in some forms of SCARs may be beneficial.

## Introduction

Severe cutaneous adverse reactions (SCARs) secondary to drugs, includes a constellation of idiosyncratic diagnoses that are associated with significant morbidity and potential mortality. SCARs include Stevens-Johnson syndrome (SJS), SJS/toxic epidermal necrolysis overlap (TEN), TEN, acute generalized exanthematous pustulosis (AGEP) and drug reaction with eosinophilia and systemic symptoms (DRESS).^1^

SJS and TEN are acute and potentially fatal mucocutaneous diseases which are largely drug induced. They are at 2 ends of a spectrum, differing only by the extent of skin detachment. In SJS, detachment is ≤10% of the skin, whereas in TEN ≥30% of skin is involved. Patients presenting with 10% to 30% skin detachment are designated SJS/TEN overlap syndrome.^2^

AGEP is characterised by an acute onset of sterile nonfollicular pustular eruption on an erythematous base starting on the face and in the folds. Mucous membrane involvement is rare.^3^^,^^4^

DRESS syndrome is characterized by fever, rash, lymphadenopathy, internal organ dysfunction, and haematological findings including eosinophilia.^5^ Mucosal lesions may occur in over 50% of cases.^6^ DRESS may be complicated by late manifestations including diabetes mellitus, cardiac disease, and thyroid disease.

## Materials and methods

Ethical approval was obtained from the Mona Campus Research Ethics Committee of the University of the West Indies (UHWI) (CREC-MN.0342, 2021/2022).

A retrospective cohort study, using chart reviews, was conducted of all patients admitted to the UHWI under the care of the dermatology specialty with a SCARs diagnosis between January 1, 2012 and June 1, 2022. The data was analyzed using STATA version 14.

## Results

There were 288 patients admitted to the UHWI dermatology specialty during the study period. Fifty-one (*n* = 51, 17.7%) of dermatology admissions had a diagnosis consistent with SCARs.

The most frequent SCARs diagnosis was TEN (*n* = 20, 39.2%) followed by AGEP with (*n* = 9, 17.6%) and DRESS (*n* = 9, 17.6%). Seven cases (*n* = 7, 13.8%) of SJS/TEN overlap were recorded and 6 cases (11.8%) of SJS. SJS, SJS/TEN overlap and TEN, accounted for 71.2% (*n* = 33) of the cases.

Females were more commonly affected in SCARs, with 64.7% (*n* = 33) of the patients being female and 35.3% (*n* = 18) being male; resulting in a F:M ratio of 1.8:1 (Table I).

**Table I:** Frequency of type of severe cutaneous adverse reaction by sex

Diagnosis	Female	Male	Total
AGEP	5	4	9
DRESS	5	4	9
SJS	6	0	6
SJS/TEN	6	1	7
TEN	11	9	20
Total	33	18	51

*AGEP*, Acute generalized exanthematous pustulosis; *DRESS*, drug reaction with eosinophilia and systemic symptoms syndrome; *SJS*, Stevens-Johnson syndrome; *TEN*, toxic epidermal necrolysis.

**Fig 1:**
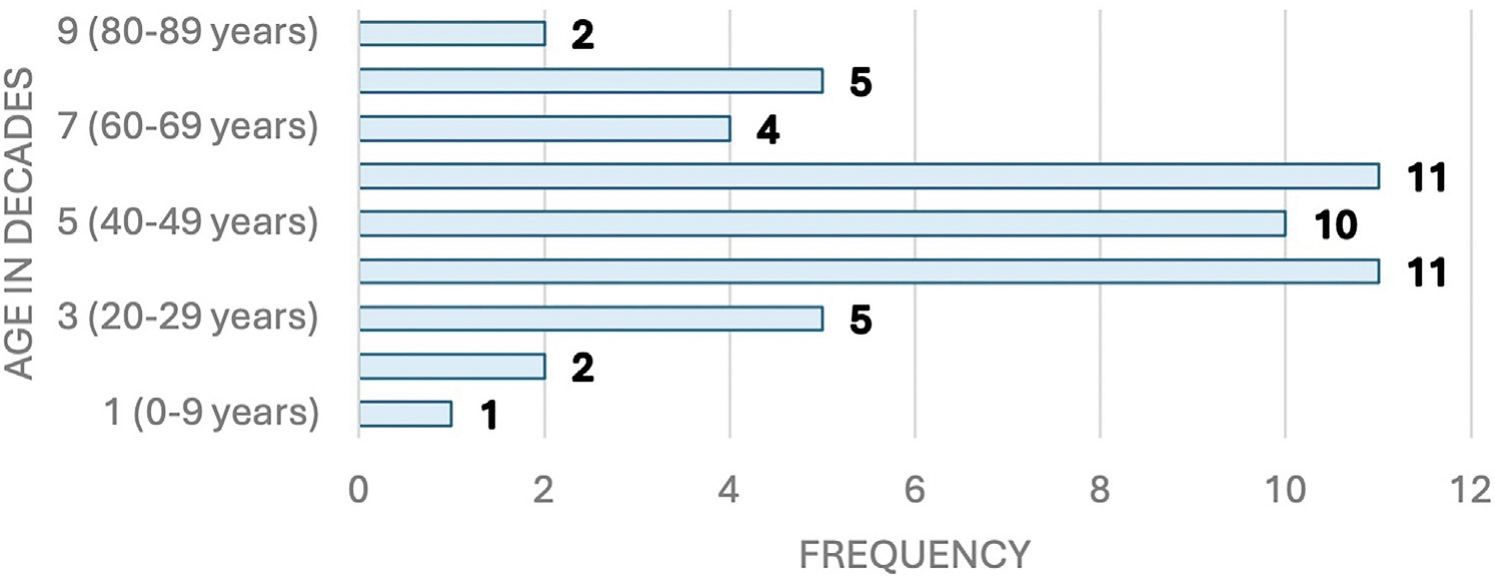
SCARs. Frequency of SCARs according to age in decade of life. *SCAR*, Severe cutaneous adverse reaction.

The age range of SCAR patients was 3-86 years. The mean age of the patients was 46.5 years (SD 18.9). The mean age for SJS was 58 years (SD 22.5), 47.9 years (SD 21.9) for SJS/TEN overlap, 38.5 years (SD 16.7) for TEN, 47.3 years (SD 18.8) in AGEP, and 55 years (SD 13.7) for DRESS. The age groups with the highest number of SCARs were the fourth, fifth, and sixth decades of life, with a total of 32 patients (62.75%) (Fig 1, Table II).

**Table II:** Frequency of each severe cutaneous adverse reaction diagnosis by decade of life

Age group	AGEP	DRESS	SJS	SJS/TEN	TEN	Total
1 (0-9)	0	0	0	0	1	1
2 (10-19)	0	0	0	0	2	2
3 (20-29)	2	0	0	1	2	5
4 (30-39)	1	1	2	2	5	11
5 (40-49	2	2	0	2	4	10
6 (50-59)	1	3	1	0	6	11
7 (60-69)	2	1	1	0	0	4
8 (70-79)	1	2	1	1	0	5
9 (80-89)	0	0	1	1	0	2
Total	9	9	6	7	20	51

*AGEP*, Acute generalized exanthematous pustulosis; *DRESS*, drug reaction with eosinophilia and systemic symptoms syndrome; *SJS*, Stevens-Johnson syndrome; *TEN*, toxic epidermal necrolysis.

**Fig 2:**
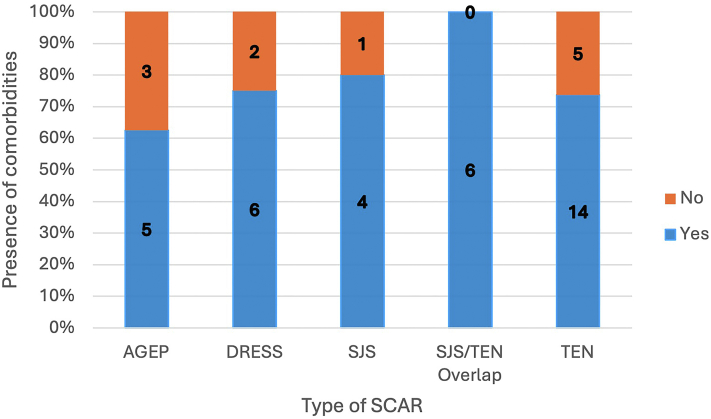
SCARs. Presence of comorbidities in each SCAR diagnosis. *SCAR*, Severe cutaneous adverse reaction.

The presence/absence of comorbidities was evaluable for 46 patients. Overall, 35 patients with SCARs were found to have comorbidities (Fig 2).

**Fig 3:**
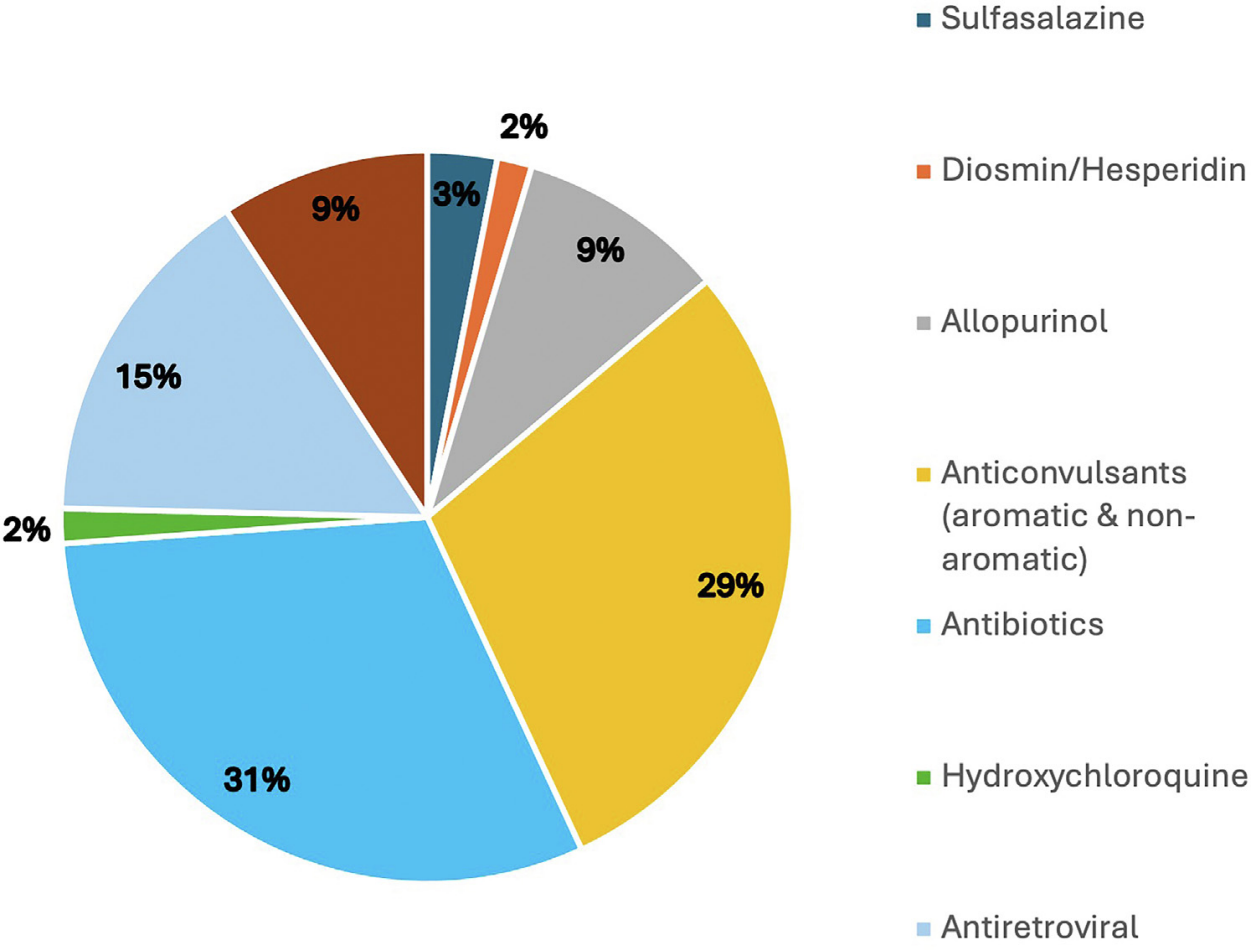
SCARs. Relative frequency of suspected causative drugs for SCARs. *SCAR*, Severe cutaneous adverse reaction.

The definitive drug information was available for 46 patients. Of those, 33 had a single suspected drug, 8 had 2 implicated drugs, 4 had 3 suspected drugs, and 1 had 4 suspected drugs. Antibiotics (*n* = 20, 31%) accounted for most of the suspected instances of SCARs followed by anticonvulsants which accounted for 29% (*n* = 19). Antiretrovirals were the suspected agents in 15% (*n* = 10) of SCARs. Allopurinol and NSAIDs/analgesics were each suspected drugs in 9% (*n* = 6) of SCARs. Diosmin/hesperidin, hydroxychloroquine, and sulfasalazine were less frequently suspected (Fig 3). Table III outlines the suspected drugs in each type of SCAR.

**Table III:** Suspected drugs in AGEP, DRESS, SJS, SJS/TEN overlap and TEN

Suspected Drug	AGEP	DRESS	SJS	SJS/TEN	TEN	Total
Acetaminophen	0	0	0	0	1	1
Allopurinol	0	1	2	1	2	6
Carbamazepine	0	3	0	0	1	4
Ceftazidime	0	0	0	0	1	1
Cefuroxime	2	0	0	0	0	2
Celecoxib	0	0	0	0	1	1
Ciprofloxacin	1	1	0	0	0	2
Clindamycin	1	0	0	0	0	1
Cotrimoxazole	0	0	1	1	3	5
Diclofenac potassium	1	0	0	0	0	1
Diosmin/hesperidin	0	0	0	1	0	1
Efavirenz	0	0	0	0	1	1
Hydroxychloroquine	0	0	0	1	0	1
Lamivudine	0	0	0	1	1	2
Levetiracetam	0	0	0	0	1	1
Levofloxacin	1	0	0	0	0	1
Meloxicam	0	1	0	0	0	1
Metamizole	0	0	0	0	2	2
Metronidazole	0	0	1	0	0	1
Nevirapine	0	0	0	2	2	4
Penicillin	4	1	0	0	1	6
Phenytoin	0	4	1	3	5	13
Sulfasalazine	0	0	0	0	2	2
Topiramate	0	0	0	1	0	1
Zidovudine	0	0	0	2	1	3
Unknown ARV	1	0	0	0	0	1

*AGEP*, Acute generalized exanthematous pustulosis; *ARV*, antiretroviral; *DRESS*, drug reaction with eosinophilia and systemic symptoms syndrome; *SJS*, Stevens-Johnson syndrome; *TEN*, toxic epidermal necrolysis.

**Fig 4:**
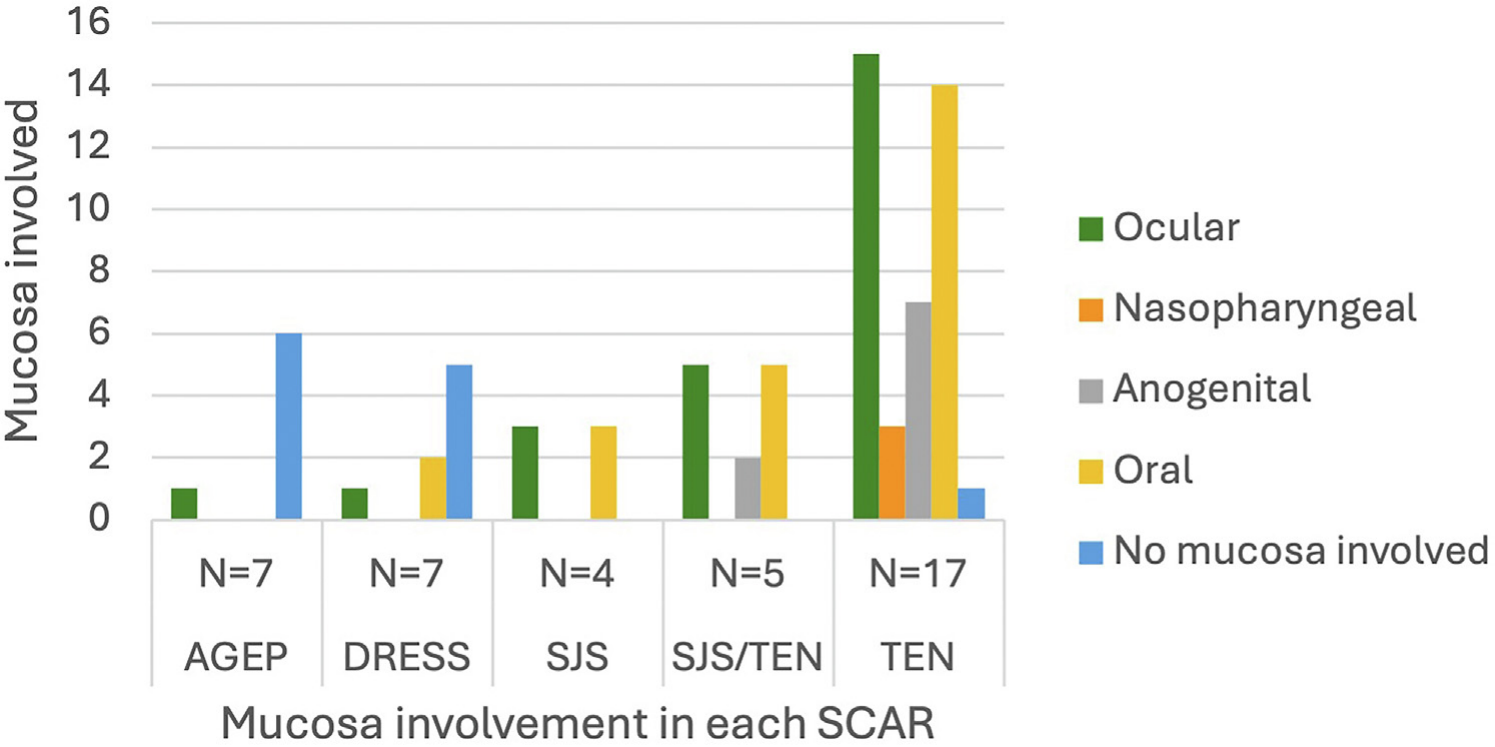
SCARs. Clustered bar showing frequency of mucosal involvement found in each SCAR diagnosis. *SCAR*, Severe cutaneous adverse reaction.

The conjunctiva was the most affected mucosa across all SCARs (*n* = 5) whereas the nasopharynx was the least affected (*n* = 2). A single patient with TEN had multiple mucosal surfaces involved (Fig 4).

Pustules and morbilliform eruptions were the predominant skin lesions seen in AGEP. Morbilliform lesions were the most common skin lesions in DRESS. Blisters, detachment and targetoid lesions were most commonly seen in the SJS-TEN spectrum (Table IV).

**Table IV:** Frequency of type of skin lesions for each severe cutaneous adverse reaction

Skin lesion	AGEP	DRESS	SJS, SJS/TEN overlap, TEN	Total
Blisters	2	0	22	24
Detachment	0	0	24	24
Erythroderma	0	1	1	2
Facial swelling	1	1	1	3
Morbilliform	6	6	11	23
Psoriasiform	0	0	0	0
Purpura	0	0	0	0
Pustules	9	0	0	9
Targetoid	1	2	15	18
Urticaria	0	0	0	0

*AGEP*, Acute generalized exanthematous pustulosis; *DRESS*, drug reaction with eosinophilia and systemic symptoms syndrome; *SJS*, Stevens-Johnson syndrome; *TEN*, toxic epidermal necrolysis.

Eosinophilia (bone marrow) and hepatic derangement were the most common organ involvement across all SCARs. The liver was the most affected organ in AGEP and SJS, SJS/TEN overlap, and TEN. In DRESS, both eosinophilia and hepatic derangement were most commonly seen.

Bacteremia and skin infections were primarily seen in the SJS spectrum. Skin infection correlated with increased amounts of skin detachment across the SJS spectrum (Table V).

**Table V:** Proportion of patients with skin infections and bacteremia in SJS, SJS/TEN Overlap and TEN

Evaluated SCAR	Skin infections	Bacteraemia
SJS (*n* = 3)	1 (33.33%)	1 (33.33%)
SJS/TEN Overlap (*n* = 5)	3 (60%)	0 (0%)
TEN (*n* = 17)	12 (70.59%)	8 (47.06%)

*SJS*, Stevens-Johnson syndrome; *TEN*, toxic epidermal necrolysis.

Acute ophthalmic complications, including visual disturbances, bacterial conjunctivitis, and dry eyes, were seen in 25 patients (49%).

Forty-eight (*n* = 48, 94.1%) patients with a SCAR were primarily managed by dermatology and 3 patients (5.9%) were primarily managed by internal medicine. Five patients (9.8%) were housed at some point in the intensive care unit or high dependency unit.

Most patients received combinations of topical and systemic corticosteroids. All AGEP cases except for 1 patient, received some form of systemic corticosteroid and all patients with DRESS received a form of systemic corticosteroid. All cases of SJS, SJS/TEN, and TEN, except for one (1) case of TEN (who had overwhelming sepsis at the time of admission), received combinations of systemic and topical corticosteroids.

All (100%) SJS/TEN overlap and TEN patients received antimicrobials (topical or systemic) of some kind. Fifty percent (50%) of SJS patients received systemic antimicrobials with topical agents. For AGEP, 66.7% patients received antimicrobial agents, all receiving a combination of topical and systemic antimicrobials. For DRESS, 70% patients received antimicrobial systemic and/or topical therapy.

Overall, the mean period of admission for SCARs was 13 days (median 10 days). The longest periods of admission were for TEN with a mean of 19 days (median 14 days) followed by DRESS with a mean of 15 days (median 10 days). For the remaining SCARs, in descending order, the mean duration of hospital admission was 10 days (median 8 days) for SJS/TEN overlap, 6 days (median 6 days) for AGEP and 6 days (median 5 days) for SJS.

Follow-up data were available for 37 patients. Long-term chronic complications affected 26 (70.3%) of these patients. Four patients had more than one chronic complication. Postinflammatory pigment alteration was the most common long-term complication representing 62.2% (*n* = 23) of the patients who had chronic complications. Cardiac (cardiomegaly)(*n* = 1), ophthalmic (xerosis and symblepharon) (*n* = 4), liver cirrhosis (*n* = 1), and steroid induced hypertension (*n* = 1) complications were seen less frequently. TEN patients had the greatest number of chronic complications, whereas AGEP had the least number of chronic complications.

Overall mortality from SCARs was 7.8%. AGEP, DRESS, and SJS/TEN overlap had 0% mortality. One patient (14%) with SJS died while 3 patients (15%) with TEN demised.

The mean age for mortality was 55 (SD 24.2). Of the 4 patients who died, 3 were female. Two (2) patients had multiple comorbidities; the comorbidities included cerebrovascular accident (*n* = 2), end-stage renal disease (*n* = 1), hypertension (*n* = 1), lower respiratory tract infection (*n* = 1), and dementia (*n* = 1). Allopurinol was implicated as the causative drug in 2 of these cases while celecoxib and acetaminophen were the suspected cause in each of the 2 remaining cases. Sepsis was the main cause of mortality (Table VI).

**Table VI:** Mortality cases, suspected drugs and sepsis in SCARs

Mortality case	Age (years)	Sex	Comorbidity	SCAR	Suspected drug	Sepsis	Positive skin culture
1	58	Male	End stage renal disease, hypertension, cerebrovascular accident	TEN	Allopurinol	Yes	Yes
2	58	Female	None	TEN	Celecoxib	Yes	Yes
3	18	Female	None	TEN	Acetaminophen	Yes	Yes
4	86	Female	Cerebrovascular accident, lower respiratory tract infection, dementia	SJS	Allopurinol	Not known (died in 24 h)	Not known

*SCAR*, Severe cutaneous adverse reaction; *SJS*, Stevens-Johnson syndrome; *TEN*, toxic epidermal necrolysis.

## Discussion

### SJS, SJS/TEN overlap, and TEN

There was a female predominance for the SJS spectrum in our study which is in keeping with previous studies.^7^ Sunaga et al also concurred with our higher incidence of these conditions in the fourth, fifth and 6th decade. The increased risk of chronic disease and drug therapy with increasing age could explain this finding.

Almost all SJS, SJS/TEN overlap, and TEN cases had involvement of the mucosa and TEN had the greatest number of mucosal sites involved simultaneously. Klimas et al had similar findings and also reported that ≥2 mucosal surfaces were typically affected.^8^

The most commonly suspected group of drugs causing TEN were aromatic anticonvulsants which contrasts with a previous study in Jamaica where antimicrobials were most commonly implicated in TEN.^9^ Kim et al also found that aromatic anticonvulsants had a 4-fold risk of SJS spectrum in addition to DRESS, when compared to nonaromatic anticonvulsants.^10^ We had 1 case of TEN secondary to acetaminophen; this has not previously been reported in Jamaica and seems to be rare worldwide.^11^

At least 75% of patients who died had skin infections and bacteraemia/sepsis concurrently. Fifty percent (50%) of those who died had at least 2 comorbidities. Hsu et al found that predictors of mortality included increasing age, increasing number of chronic health conditions, infection, haematological malignancy, and renal failure (*P* ≤ .03 for all).^12^

The use of corticosteroids is an important part of our treatment regime as shown in this study. In a study in Japan of the SJS-TEN spectrum, the actual mortality rate (7%) was lower than the predicted mortality rate (21.6%) in the corticosteroid pulse group. They also found that in the surviving patients there was an absence of ocular sequelae when treated with corticosteroids.^13^

### AGEP and DRESS

Both DRESS and AGEP had a slight female predominance in our study and this has been confirmed in other studies.^14^^,^^15^ Our study found that the most frequently implicated drugs in AGEP were antibiotics with penicillin being the most common; this concurs with other research where beta lactam antimicrobials were most implicated.^14^

One case of AGEP in our study (14.3%) had mucosal involvement with ocular lesions. This contrasts with reports of mucosal involvement occurring in 20% to 25% of patients with AGEP, primarily the oral mucosa.^16^ We found that the most frequently involved organ in AGEP was the liver with no simultaneous multiorgan involvement, although multiorgan involvement has been reported in up to 17% of cases.^17^ In 14.3% of our cases, eosinophilia occurred compared to 30% in similar studies.^16^^,^^17^

Our average length of hospitalization for AGEP with a mean of 6.2 days (median = 6.6) was lower than other studies where median length of hospitalization was 10 days.^18^ Eighty-three percent (83%) of our patients with AGEP received systemic corticosteroids and retrospective AGEP studies have noted reduction in the length of hospitalization with systemic corticosteroid treatment (*P* = .035).^18^

We observed a mortality rate of 0% for AGEP but mortality rates have been reported of approximately 5%, usually seen in patients with previous existing comorbid diseases.^19^

Literature confirms that anticonvulsants, usually aromatic forms, are highly implicated in DRESS as we found in our study.^20^ Eosinophilia has been reported as the most frequently occurring haematological abnormality (>50% of cases)^5^ which was in keeping with our findings of eosinophilia in 100% of our cases of DRESS.

We had 0% mortality for DRESS, contrasting with published literature which describes mortality rates up to 10%.^21^ Thrombocytopenia, chronic renal insufficiency, multi-organ involvement, pancytopenia, tachypnoea, tachycardia, coagulopathy, gastrointestinal bleeding, and leucocytosis are frequently associated with fatal outcomes.^22^^,^^23^ Mucosal surfaces are usually spared in DRESS^24^ but we found that 28.6% of our patients had mucosal involvement.

The main limitation of this study was that it was performed at a single center in Jamaica and, therefore, the results may not be generalizable. Also, as the study was retrospective there were missing data and some patients were lost to follow up.

## Declaration of Generative AI and AI-assisted technologies in the writing process

No Generative AI was used in the preparation of this manuscript.

## Conflict of interest

None disclosed
